# Preclinical Molecular PET-CT Imaging Targeting CDCP1 in Colorectal Cancer

**DOI:** 10.1155/2021/3153278

**Published:** 2021-09-13

**Authors:** Tahleesa J. Cuda, Yaowu He, Thomas Kryza, Tashbib Khan, Brian W. Tse, Kamil A. Sokolowski, Cheng Liu, Nicholas Lyons, Madeline Gough, Cameron E. Snell, David K. Wyld, Stephen Rose, Andrew D. Riddell, Andrew R. L. Stevenson, Paul A. Thomas, David A. Clark, Simon Puttick, John D. Hooper

**Affiliations:** ^1^Faculty of Medicine, The University of Queensland, Brisbane, QLD, Australia; ^2^Mater Research Institute The University of Queensland, Translational Research Institute, Woolloongabba, QLD, Australia; ^3^Royal Brisbane and Women's Hospital, Metro North Hospital and Health Service, Brisbane, QLD, Australia; ^4^Preclinical Imaging Core Facility, Translational Research Institute, Woolloongabba, QLD, Australia; ^5^Envoi Specialist Pathologists, Herston, QLD, Australia; ^6^QIMR Berghofer Medical Research Institute, Herston, QLD, Australia; ^7^Mater Pathology, Mater Hospital Brisbane, Mater Health Services, Brisbane, QLD, Australia; ^8^Commonwealth Scientific and Industrial Research Organisation, Herston, QLD, Australia; ^9^Redcliffe Hospital, Metro North Hospital and Health Service, Brisbane, QLD, Australia

## Abstract

Colorectal cancer (CRC) is the third most common malignancy in the world, with 22% of patients presenting with metastatic disease and a further 50% destined to develop metastasis. Molecular imaging uses antigen-specific ligands conjugated to radionuclides to detect and characterise primary cancer and metastases. Expression of the cell surface protein CDCP1 is increased in CRC, and here we sought to assess whether it is a suitable molecular imaging target for the detection of this cancer. CDCP1 expression was assessed in CRC cell lines and a patient-derived xenograft to identify models suitable for evaluation of radio-labelled 10D7, a CDCP1-targeted, high-affinity monoclonal antibody, for preclinical molecular imaging. Positron emission tomography-computed tomography was used to compare zirconium-89 (^89^Zr)-10D7 avidity to a nonspecific, isotype control ^89^Zr-labelled IgG*κ*1 antibody. The specificity of CDCP1-avidity was further confirmed using CDCP1 silencing and blocking models. Our data indicate high avidity and specificity for of ^89^Zr-10D7 in CDCP1 expressing tumors at. Significantly higher levels than normal organs and blood, with greatest tumor avidity observed at late imaging time points. Furthermore, relatively high avidity is detected in high CDCP1 expressing tumors, with reduced avidity where CDCP1 expression was knocked down or blocked. The study supports CDCP1 as a molecular imaging target for CRC in preclinical PET-CT models using the radioligand ^89^Zr-10D7.

## 1. Introduction

Colorectal cancer (CRC) is the third most common malignancy and the fourth leading cause of cancer-related death in the world [1]. Metastatic disease is the major cause of death with liver and lung the most common sites of metastasis in 50–60% and 10–30% of CRC patients, respectively [2–5]. Staging and assessment of treatment response are aided by imaging using contrast-enhanced chest, abdominal and pelvic computed tomography (CT) with or without magnetic resonance (MR) and 2-deoxy-2-[F-18] fluoroglucose (FDG) positron emission tomography (PET)-CT for improved anatomical localisation, lesion differentiation, and detection of small lesions [4, 6, 7].

Cancer diagnosis, risk stratification, therapy prognostication, and assessment of treatment efficacy can be improved by additional imaging approaches that noninvasively characterise and measure biological processes *in vivo* at the molecular level [8]. Molecular imaging integrates 2D or 3D imaging with cumulative quantification of cellular events using a variety of protocols including nuclear medicine radioligand imaging, MR imaging, MR spectroscopy, optical imaging, and ultrasound [9]. Radioligand molecular imaging employs ligands that incorporate a radionuclide conjugated to a peptide or antibody component that is specific for a protein enriched on the surface of malignant cells [10]. Systemically administered radioligands locate and bind to tumors and emit a radioactive signal to allow real-time detection using nuclear imaging modalities, such as PET, enabling determination of a range of clinically important parameters such as antigen biodistribution, anatomical location, pharmacokinetics, response to therapy, dose thresholds for malignant lesions, and off-target dosimetry [10–18]. As such, radioligand molecular imaging is a valuable tool for disease staging and guiding treatment decision making [2, 19, 20]. When combined with CT or MRI, both cellular and morphological features are acquired simultaneously [13].

An effective target protein for radioligand molecular imaging is expressed on the surface of tumor cells to be accessible to systemically delivered radioligands. To attain a high tumor-to-normal tissue ratio, candidate proteins should have homogeneous tumor expression with limited expression in normal tissue [17, 21]. Complement C1r/C1s, Uegf, Bmp1 domain-containing protein-1 (CDCP1) is a type I membrane-spanning glycoprotein with a 636 amino acid extracellular region, 20 amino acid transmembrane region, and 150 amino acid cytoplasmic domain [22, 23]. It is also known as subtractive immunisation M^+^ HEp3-associated 135 kDa protein (SIMA135), Transmembrane and Associated with Src Kinases (TRASK), and cluster of differentiation 318 (CD318) [22, 23]. CDCP1 is expressed as a full-length 135 kDa protein and can also undergo proteolytic cleavage generating a 70 kDa membrane-spanning carboxyl-terminal fragment and a 65 kDa aminoterminal fragment that is either shed from the cell surface or remains bound to CDCP1 on the plasma membrane [24]. Previous studies have demonstrated the utility of mouse monoclonal 10D7 antibody that binds to the CDCP1 amino terminal, for delivery of Zirconium-89 (^89^Zr) for PET-CT-based detection, and cytotoxins for treatment of preclinical models of ovarian [25] and pancreatic [24] cancer. In CRC, elevated CDCP1 correlates with poorer patient outcome [3, 26]. Analysis of CDCP1 mRNA in a CRC cohort of 101 patients indicated that elevated levels correlate significantly with advanced stage, node metastasis, and diminished recurrence-free and overall survival [26]. Similarly, immunohistochemical analysis of 128 CRCs, including 38 cases without metastasis on presentation, 51 with liver metastasis, 35 with lung metastasis, and four with both liver and lung metastasis, elevated CDCP1 correlated significantly with tumor size, grade and stage, and decreased lung metastasis free survival [3].

The present study aimed to investigate CDCP1 as a potential radioligand molecular imaging target for PET-CT detection of CRC in cell line xenograft and patient-derived mouse models using ^89^Zr-labelled 10D7. The specificity of ^89^Zr-10D7 for CDCP1 expressing CRC is explored using unlabelled 10D7 to compete for antibody binding sites and via silencing of CDCP1 expression to reduce the number of antibody binding sites.

## 2. Materials and Methods

### 2.1. Cell Lines and Culture Conditions

Human CRC HCT116, HT29 and SW480, prostate cancer PC3, and ovarian cancer OVMZ6 cell lines were obtained from ATCC (Manassas, VA). PC3 cell lines were maintained in RPMI 1640. CRC HCT116, HT29 and SW480, and ovarian cancer OVMZ6 cell lines were maintained in Dulbecco's Modified Eagle's Medium Media (Thermo Fisher Scientific, Seventeen Mile Rocks, Australia) containing 10% fetal calf serum (FCS) (Sigma-Aldrich, North Ryde, Australia), penicillin (100 units per mL), and streptomycin (100 units per mL) (Sigma-Aldrich) at 37°C in a humidified, 5% CO_2_ atmosphere.

### 2.2. Silencing of CDCP1 Expression

CDCP1 expression was stably reduced in cells grown as spheroids isolated from a previously generated patient-derived xenograft (PDX), designated CRC13, as previously described [27, 28]. Cells from CRC13 spheroids mechanically dissociated in phosphate-buffered saline (PBS) containing EDTA (0.48 mM) were stably infected with a pLKO.1 lentiviral CDCP1 targeting shRNA (target sequence: GCTCATAAGAGCATCGGTTTA; Open Biosystems, Millennium Science, Surrey Hills, Australia), or as a control, a nontargeting (control) shRNA (Addgene, Watertown, MA, USA) construct. Stably infected cells were grown as spheroids and selected in media containing puromycin (2 µg/ml) generating cells designated CRC13-shScr and CRC13-shCDCP1 which were propagated as subcutaneous tumors in mice as described below.

### 2.3. Western Blot Analysis

Whole cell lysates were collected in RIPA buffer (Sigma-Aldrich) containing 1x complete protease inhibitor cocktail (Roche, Castle Hill, NSW, Australia), 10 mM sodium fluoride, and 2 mM sodium vanadate. Lysates were used in western blot analysis as previously described [29] with rabbit anti-CDCP1 carboxyl-terminal antibody #4115 (dilution 1 : 2,000; Cell Signaling Technology, Genesearch, Arundel, Australia).

### 2.4. Flow Cytometry

At 50% confluence, adherent cells were nonenzymatically detached in PBS/EDTA (0.48 mM) and counted. Cells (2.5 × 10^5^) were washed twice in PBS, blocked in PBS containing 2% FCS for 30 minutes, and stained with mouse anti-CDCP1 aminoterminal antibody 10D7 for 1 hour at 4°C [25]. Cells were then washed twice in PBS containing 2% FCS for 30 minutes at 4°C before incubation with an Alexa Fluor 488-conjugated goat anti-mouse secondary antibody at 4°C for 30 minutes (Thermo Fisher Scientific). Cells were washed in PBS twice more, then events (20,000/condition) were recorded and analysed using a FACs Fortessa flow cytometer.

### 2.5. Subcutaneous Mouse Xenograft Models

Experiments involving mice were approved by the University of Queensland Animal Ethics Committee (approval 112/17). Mice were housed in a pathogen-free environment with food and water provided ad libitum. Eight-week-old male NOD Cg-Prkdc^scid^IL2rg^tm1Wjl^/SzJ (NSG) mice (The Jackson Laboratory, Bar Harbor, ME) were injected subcutaneously with HCT116 cells (1 × 10^6^) or spheroids of CRC13, CRC13-shScr or CRC-shCDCP1 cells (0.1 g/mouse of pelleted cell slurry in PBS suspension). Tumors were grown for three weeks prior to the commencement of radioligand molecular imaging experiments.

### 2.6. Radiochemistry

Conjugation of 1-(4-isothiocyanatophenyl)-3-[6,17-dihydroxy-7,10,18,21-tetraoxo-27-(N-acetylhydroxylamino)-6,11,17,22-tetraazaheptaeicosine] thiourea (DFO-NCS) to 10D7 was performed as previously described [30]. Briefly, DFO-NCS (70 *μ*g; ∼93 nM) dissolved in dimethyl sulfoxide(5 *μ*L) was reacted with 10D7 or isotype matched control IgG*κ*1 (1.5 mg; 10 nM) in 0.1 M Na_2_CO_3_ (0.5 mL) for one hour at 37°C and pH 8.8–9.0. The reaction mixture was filtered by size exclusion chromatography using an AKTA Prime Plus (GE Life Sciences) fitted with a 5 mL HiTrap desalting column running a mobile phase of 0.1 M PBS at 0.5 mL/min. Radio labelling of DFO conjugated antibodies with ^89^Zr was performed in accordance with published methods [30]. The pH of ^89^Zr-oxalate (Perkin Elmer, Rowville, Australia) was adjusted to pH 7.0 using aliquots of 1M Na_2_CO_3_ and buffered to pH 7.5 by 1 : 1 dilution in 0.5MHEPES. DFO-10D7 and DFO-IgG*κ*1 (1 mg) in 400 *μ*L 0.5 M HEPES (pH 7.5) was mixed with 60MBq ^89^Zr solution and incubated at 25°C for 60 minutes with mild agitation. Purification was then performed using a 7 kDa MWCO Zeba desalting column (Thermo Fisher Scientific) equilibrated with 0.1 M PBS (pH 7.2), yielding ∼49MBq of ^89^Zr-DFO-10D7 and ^89^Zr-DFO-IgG*κ*1 in 0.5 mL (radiochemical yield 81%; concentration98MBq/mL and 49MBq/mg). Thin-layer chromatography confirmed radiochemical purity >95% [30].

### 2.7. Radioligand Biodistribution Analysis

Mice (*n* = 4 per group) were anaesthetised with isoflurane and injected intravenously (i.v.) via lateral tail vein. For experiments using HCT116, mice were injected with one of the following regimes: (1) ^89^Zr-10D7 (^89^Zr 1.4 MBq; 23 *µ*g 10D7), (2) unlabelled 10D7 (1 mg) [31] after 60 minutes followed by ^89^Zr-10D7 (^89^Zr 1.4 MBq; 23 *µ*g 10D7), or (3) ^89^Zr-IgG1*κ* (^89^Zr 1.4 MBq; 23 *µ*g IgG1*κ*). Radioligand dosing was based on recent published data with increased dose provision to lower CDCP1 tumor types [24]. For experiments using CRC13, ^89^Zr-10D7 (^89^Zr 2 MBq; 33 *µ*g 10D7), unlabelled 10D7 (1 mg) [31], followed after 60 minutes by ^89^Zr-10D7 (^89^Zr 2 MBq; 33 *µ*g 10D7) or ^89^Zr-IgG1*κ* (^89^Zr 2 MBq; 33 *µ*g IgG1*κ*). PET-CT imaging was performed at 1, 24, 48, 72, and 144 hours following radioligand administration using an Inveon PET-CT system (Siemens, Munich, Germany). Thirty-minute PET image acquisition was performed. CT imaging (10 minute acquisition) was performed for anatomical registration and attenuation correction (80 kV, 500 *μ*A, 230 ms exposure time, 360° rotation with 180 rotation steps, binning factor of 4, low magnification position—producing an effective pixel size of 106 *μ*m). CT images were reconstructed using the Feldkamp algorithm. PET images were reconstructed using an ordered subset expectation maximisation (OSEM2D) algorithm with CT attenuation correction. A conversion factor obtained from a cylindrical phantom filled with a known activity of ^89^Zr was used to convert PET activity per voxel to becquerel (bq)/cubic centimetre (cc). Image reconstruction and data analysis were performed using the Inveon Research Workspace (Siemens). *In vivo* tissue radioactivity was analysed at each time point within regions of interest (ROI) recorded as the percent of injected dose per unit volume (cubic centimetre) of tissue (%ID/cc). During measurement of each tumor deposit or organ, careful segmentation was performed only inclusive of the ROI. *Ex vivo* biodistribution of radioligand signal was performed after the 144-hour imaging time point. Mice were euthanised by CO_2_ asphyxiation and a blood sample obtained by heart puncture. Tissues (tumor, heart, lungs, liver, kidneys, femur, muscle, tail, blood, and testes) were harvested and weighed. Radioactivity of ROIs was measured in a Wizard 2480 Automatic Gamma Counter (PerkinElmer) as the percent of the injected dose per unit weight (*g*) of tissue (%ID/g) calculated was corrected for decay and detector efficiency.

### 2.8. Immunohistochemical Analysis

Tumors were fixed in 10% neutral buffered formalin (Sigma-Aldrich) and then embedded in paraffin. Deparaffinised and rehydrated sections (5 *µ*m) were stained with rabbit anti-CDCP1 carboxyl-terminal antibody #4115 (1 : 100) and haematoxylin and eosin (H&E) as previously described with signal developed using immunoperoxidase with DAB (DakoCytomation, Glostrup, Hovedstaden, Denmark) as the chromogenic substrate [29]. Sections were imaged with an Olympus DP26 camera and associated cellSens standard 1.7 imaging software (Olympus, Notting Hill, Australia). Staining was assessed by FRCPA anatomical pathologists (CL, CES).

### 2.9. Statistical Analysis

Statistical analyses were performed using GraphPad Prism 8 (GraphPad Software, San Diego, CA, USA). Statistical analyses of *in vivo* and *ex vivo* datasets were carried out individually, using two-tailed Student's *t*-test and two-way ANOVA analysis of variance. Values represent the mean ± standard deviation (SD). A value of *p* < 0.05 was considered statistically significant. This paper was written in accordance with the ARRIVE guidelines [32].

## 3. Results

### 3.1. Cell-Based and Mouse Xenograft Assays Identify HCT116 Cells as Suitable for Assessment of CDCP1-Directed Radioligand Molecular Imaging for CRC

**Figure 1:**
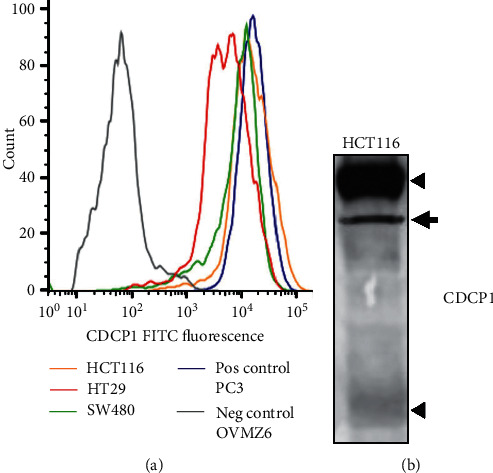
Cell-based and mouse xenograft assays identify HCT116 cells for assessment of CDCP1-directed radioligand molecular imaging for CRC. (a) Flow cytometry evaluation of cell surface CDCP1 expression on HCT116, SW480, and HT29 CRC cell lines, positive control prostate cancer PC3 cells, and negative control ovarian cancer OVMZ6 cells. Cells were stained with anti-CDCP1 antibody 10D7 then Alexa Fluor 488 tagged goat-anti-mouse secondary antibody. Cells stained only with the secondary antibody provided background signal. Signals were normalised to mode. (b) Western blot analysis for CDCP1 of lysates from HCT116 cells using anti-carboxyl terminal antibody 4115. Arrowheads indicate full-length 135 kDa and cleaved 70 kDa CDCP1. An incompletely N-glycosylated form of CDCP1 is indicated by the arrow.

To identify a cell line suitable for CDCP1-targeted radioligand molecular imaging, flow cytometry assessing cell surface expression of CDCP1 was performed on the three CRC lines HT29, SW480, and HCT116, with ovarian cancer OVMZ6 cells used as a negative control and prostate cancer PC3 cells as a positive control [25,33]. As shown in Figure 1(a), cell surface levels of CDCP1 were the highest on HCT116 and SW480 cells, comparable to levels on prostate cancer PC3 cells, while levels were at least 50% lower on HT29 CRC cells. Western blot analysis of cell lysates indicated that CDCP1 is predominantly expressed by HCT116 cells as the full-length 135 kDa protein with only low levels of the 70 kDa carboxyl-terminal fragment generated by proteolysis (Figure 1(b)). High levels of primarily full-length CDCP1 retained on the plasma membrane should act as a suitable candidate for radioligand molecular imaging of HCT116 cell xenografts in mice.

### 3.2. CDCP1-Targeted Molecular PET-CT Imaging Detects a CRC Cell Line Xenograft

**Figure 2:**
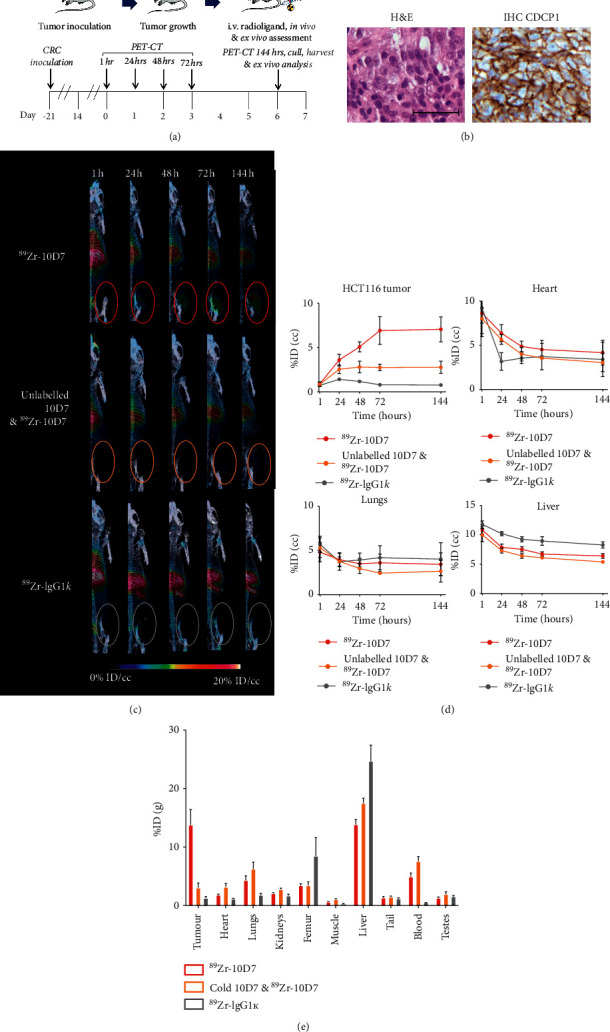
CDCP1 targeted molecular PET-CT imaging detects a CRC cell line xenograft. (a) Diagram of HCT116 cell inoculation followed by a period of tumor growth, radioligand administration PET-CT imaging. (b) Representative H&E staining (left) and anti-CDCP1 immunohistochemical staining (right) demonstrating strong plasma membrane staining of CDCP1 on malignant cells in subcutaneous HCT116 cell xenograft tumors in mice (magnification 40X; scale bar 50 *μ*m). (c) Representative 3D PET-CT imaging reconstructions of mice bearing subcutaneous HCT116 cell xenografts administered i.v.^89^Zr-10D7, unlabelled 10D7 with ^89^Zr -10D7, or ^89^Zr-IgG1*κ* (1.4MBq) at 1, 24, 48, 72, and 144 hours after administration. (d) Graph of the time course of *in vivo* PET avidity of HCT116 cell xenograft tumor, heart, lungs, and liver at 1, 24, 48, 72, and 144 h after i.v. administration of ^89^Zr-10D7, unlabelled 10D7 with ^89^Zr -10D7, and ^89^Zr-IgG1*κ*. Error bars are present for ^89^Zr-IgG1*κ* but at the scale are too small to be apparent. (e) Graph of radioactivity of recovered tumors measured by *ex vivo* radiometric analysis and displayed as %ID/g. ^*∗∗*^, *p* < 0.01.

HCT116 cells grown as subcutaneous tumors in mice were employed to assess the ability of CDCP1-targeted molecular imaging to detect CRC *in vivo*. As summarized in Figure 2(a), after three weeks of HCT116 cell growth as xenografts, mice were injected i.v. with ^89^Zr-10D7, unlabelled 10D7 followed by^89^Zr-10D7 after 60 minutes, or control ^89^Zr-IgG1*κ*, and PET-CT imaging was performed at 1, 24, 48, 72, and 144 hours later. Histological analysis of representative untreated subcutaneous HCT116 tumors revealed that xenografts display adenocarcinoma histological features (Figure 2(b) left) and CDCP1 expression which is located predominantly on the surface of malignant cells (Figure 2(b) right). These data confirm that HCT116 cell xenografts are suitable for assessment of CDCP1-directed molecular imaging of CRC *in vivo*.

*In vivo* PET analysis indicated that ^89^Zr-10D7 signal increased up to 72 hours then plateaued (Figures 2(c) and 2(d); statistical analysis in Table S1). It revealed that accumulation of ^89^Zr-10D7 in tumors (7.1 ± 1.4%ID/cc at 144 hours) was largely ablated by competition with unlabelled 10D7 (2.8 ± 0.7%ID/cc at 144 hours), while mice injected with control ^89^Zr-IgG1*κ* had negligible tumor avidity (0.8 ± 0.1%ID/cc at 144 hours). This analysis also demonstrated that in contrast to increasing ^89^Zr-10D7 signal in tumor sup to 72 hours, off-tumor ^89^Zr signal in heart, lungs, and liver rapidly decreased in the first 24 hours and then plateaued in all groups of mice (Figure 2(d)).

At the end of the assay, quantitative radiometric gamma counter analysis was performed to further examine tumor and off-tumor radioactivity. The data are provided in Table S2, and statistically significant differences are shown in Table S3. The results demonstrate that endpoint radioactivity in tumors was 11.2 fold higher in mice administered ^89^Zr-10D7 than ^89^Zr-IgG1k (Figure 2(e); Table S2) confirming the CDCP1-specific binding and uptake apparent from the PET analysis. The specificity of 10D7 for CDCP1 expressing CRC cells *in vivo* was further confirmed by results showing that signal from xenograft tumors was 78% lower in mice coadministered unlabelled 10D7 and ^89^Zr-10D7 compared with mice administered only ^89^Zr-10D7 (Figure 2(e); Table S1).

Examination of off-tumor signal by *ex vivo* radiometric gamma counter analysis activity demonstrated the significantly higher hepatic activity of ^89^Zr-IgG1*κ* in comparison to ^89^Zr-10D7 coadministered with unlabelled 10D7, followed by ^89^Zr-10D7. It also revealed relatively lower blood, heart, lung, and renal activity of ^89^Zr-IgG1*κ* compared to ^89^Zr-10D7 coadministered with unlabelled 10D7 followed by ^89^Zr-10D7 (Figure 2(e)). These findings may be a consequence of reduced specific binding of ^89^Zr-IgG1*κ* resulting in increased hepatic uptake and catabolism as a mononuclear phagocyte system-containing organ [34–38]. Furthermore, high femoral activity was also identified in mice that received ^89^Zr-IgG1*κ* (Figure 2(e)). This finding further supports this hypothesis of increased ^89^Zr-IgG1*κ* hepatic catabolism with unbound ^89^Zr accumulating within unfused skeletal epiphyses of the skeletally immature mice used in these experiments, a known ^89^Zr phenomenon [39, 40]. High blood activity was detected in mice that received unlabelled 10D7 combined with ^89^Zr-10D7 (7.5 ± 0.8%ID/g) compared to ^89^Zr-10D7 and ^89^Zr-IgG1*κ* (5.0 ± 0.6%ID/g and 0.5 ± 0.0%ID/g, respectively). We speculate that unlabelled 10D7 hepatic uptake and catabolism delayed the degradation of ^89^Zr-10D7, resulting in greater free circulating activity within the blood at 144 hours. Statistically significant differences in *ex vivo* activity are provided in Table S3.

### 3.3. CDCP1-Targeted Molecular PET-CT Imaging Detects a CRC PDX

**Figure 3:**
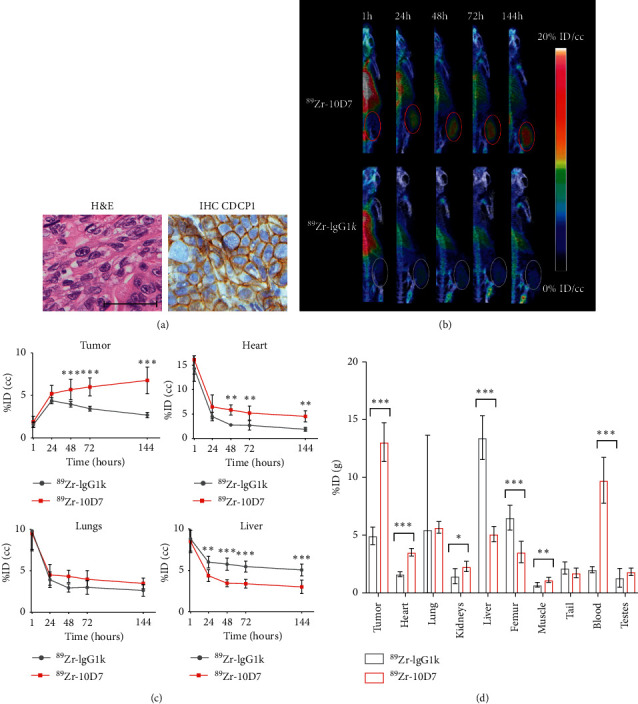
CDCP1-targeted molecular PET-CT imaging detects CDCP1-expressing patient-derived CRC13 model in mice. (a) Representative image of H&E stained (left) and anti-CDCP1 immunohistochemical staining (right) demonstrating moderate plasma membrane staining of CDCP1 on malignant cells in subcutaneous CRC13 xenograft tumors in mice (magnification 40X; scale bar 50 *μ*m_. (b) Representative 3D PET-CT imaging reconstructions of mice bearing subcutaneous CRC13 tumors administered i.v.^89^Zr-10D7 or ^89^Zr-IgG1*κ* (1MBq) at 1, 24, 48, 72, and 144 h after administration. (c) Graph of the time course of *in vivo* PET avidity of CRC13 tumors at 1, 24, 48, 72, and 144 h after i.v. administration of ^89^Zr-10D7 and ^89^Zr-IgG1*κ*. (d) Graph of radioactivity of recovered tumors, organs, and blood by *ex vivo* radiometric analysis and displayed as %ID/g. ^*∗*^, *p* < 0.05; ^*∗∗*^, *p* < 0.01; ^*∗∗∗*^, *p* < 0.001.

To assess the effectiveness of a CDCP1-targeted agent against a more disease relevant model, PET-CT imaging was performed on PDX CRC13 [28]. This patient-derived model retains the features of the original primary colon cancer displaying histology of a poorly differentiated adenocarcinoma (Figure 3(a) left) and prominent cell surface localisation of CDCP1 (Figure 3(a) right). After three weeks of CRC13 growth, PET-CT imaging of mice at 1, 24, 48, 72, and 144 hours after i.v. administration of radio-labelled antibodies indicated increasing accumulation of ^89^Zr-10D7 signal in subcutaneous flank tumors with negligible tumor signal from mice injected with control ^89^Zr-IgG1*κ* (Figure 3(b)). As was observed for the HCT116 cell xenograft model, both ^89^Zr-10D7 and ^89^Zr-IgG1*κ* accumulated in liver and the cardiopulmonary system with signal reducing significantly 24 hours after administration by *in vivo* PET analysis (Figure 3(c)). Quantitative analysis of signal from tumor, heart, lung, and liver indicated increasing ^89^Zr-10D7 tumor avidity over the time course and the highest avidity at the 144-hour time point (6.8 ± 1.6%ID/cc) whereas signal from heart, lung, and liver reduced during this time period (Figure 3(c)). These results were confirmed by quantitative *ex vivo* biodistribution analysis of ^89^Zr-10D7 and ^89^Zr-IgG1*κ* in recovered tumors, organs, and blood. As shown in Figure 3(d), markedly higher activity was detected in tumors from mice administered ^89^Zr-10D7 (13.1 ± 1.7%ID/g) compared to ^89^Zr-IgG1*κ* (4.9 ± 0.8%ID/g). Radioactivity was approximately the same in lung, tail, and testes from these mice, and levels were higher in liver and femur of mice administered ^89^Zr-IgG1*κ*, while levels were marginally higher in heart, kidney, and muscle of mice administered ^89^Zr-10D7 (Figure 3(d) and Table S4). Surprisingly, signal was considerably higher in the blood of mice administered ^89^Zr-10D7 compared with ^89^Zr-IgG1*κ* (Figure 3(d) and Table S4) likely as a result of reduced nonspecific accumulation of ^89^Zr-10D7 in off-target tissues resulting in increased, free circulating ^89^Zr-10D7 compared to ^89^Zr-IgG1*κ*. Overall findings of this experiment correlate with those from the HCT116 CRC model presented earlier.

**Figure 4:**
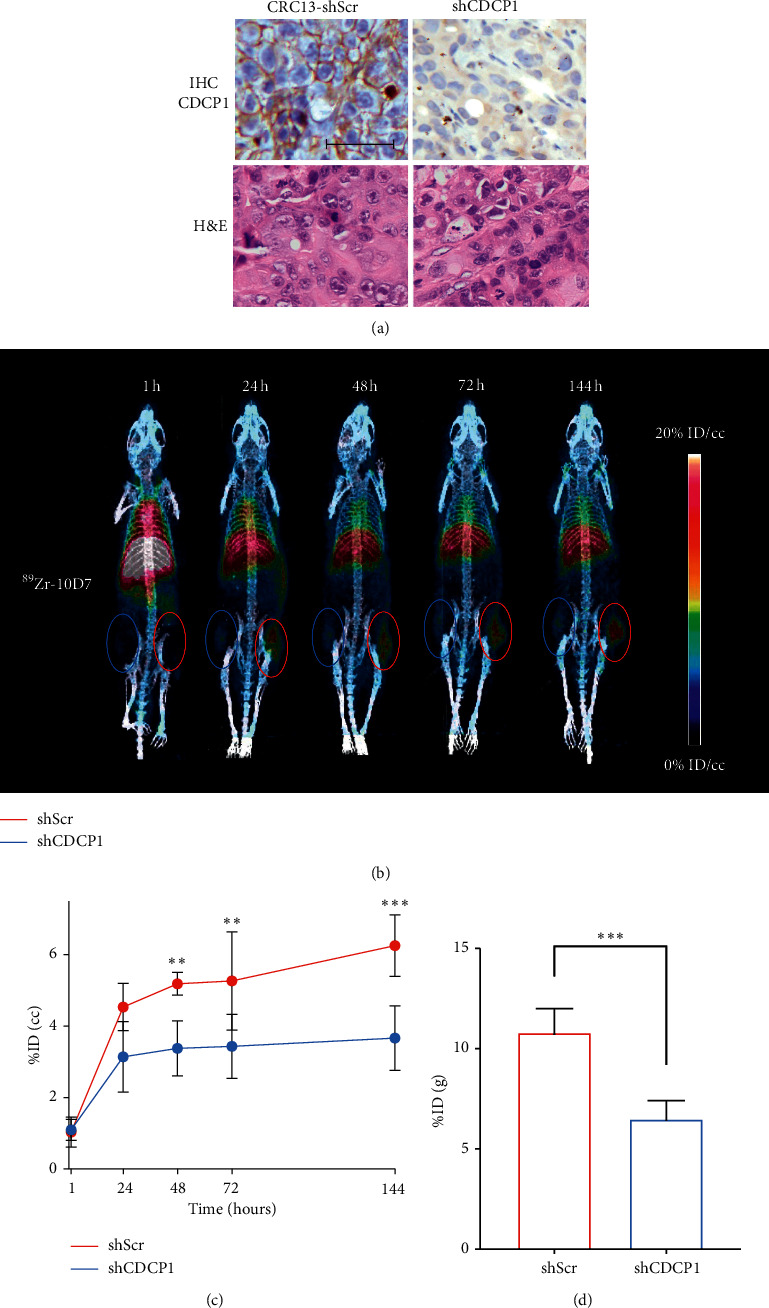
Reduced CDCP1 expression reduces avidity of CDCP1-targing antibody 10D7 for patient-derived model CRC13 in mice. (a) Representative image of H&E stained (bottom) and anti-CDCP1 immunohistochemical staining (top) of subcutaneous CRC13-shScr (left) and CRC13-shCDCP1 (right) xenografts in mice. Anti-CDCP1 immunohistochemistry demonstrated markedly reduced expression of CDCP1 in CRC13-shCDCP1 tumors compared with CRC13-shScr tumors. H&E staining indicated that histology of xenografts was unaltered by reduced levels of CDCP1 (scale bar 50 *μ*m). (b) Representative 3D PET-CT reconstructions of mice bearing CRC13-shScr (red circle) and CRC13-shCDCP1 (blue circle) tumors at 1, 24, 48, 72, and 144 hours after ^89^Zr-10D7 administration (1MBq). (c) *In vivo* PET avidity of tumors at time points following ^89^Zr-10D7 administration displayed as %ID/cc. (d) Quantitative *ex vivo* biodistribution data determined by radiometric analysis of excised tumors at the end of the assay. ^*∗*^, *p* < 0.05; ^*∗∗*^, *p* < 0.01; ^*∗∗∗*^, *p* < 0.001.

To examine the impact of reduced CDCP1 expression on ^89^Zr-10D7 tumor avidity, PET-CT imaging results were compared from CRC13 xenografts stably transduced with a lentivirus CDCP1 silencing construct (CRC13-shCDCP1) or a scramble control construct (CRC13-shScr). As shown in Figure 4(a), histochemical analyses indicated that CDCP1 expression was markedly reduced in CRC13-shCDCP1 compared to CRC13-shSrc tumors (top) and the histology of xenografts was unaltered by reduced levels of this protein (bottom). Twice weekly measurement of xenograft volume indicated that tumor growth was unaffected by silencing of CDCP1. PET-CT imaging of mice administered ^89^Zr-10D7 indicated that after 24 hours, CRC13-shCDCP1 tumors (blue circle) with reduced levels of CDCP1 had significantly lower avidity than control CRC13-shScr tumors (red circle) and the difference in avidity increased over time (Figure 4(b)). Quantitative imaging and radiometric analyses indicated that ^89^Zr-10D7 tumor avidity was reduced by about 40% in CRC13-shCDCP1 (6.5 ± 1.0%ID/g) compared to CRC13-shSrc (10.8 ± 1.3%ID/g) tumors (Figures 4(c) and 4(d)). Although IHC analysis confirmed nearly 100% downregulation of CDCP1 expression in CRC13-shCDCP1 compared to CRC13-shSrc tumors, tumor avidity varied by 40%.

## 4. Discussion

Using cell line xenograft and patient-derived models in mice, our data indicate that the radio-labelled antibody agent, ^89^Zr-10D7, directed against the receptor CDCP1, can be employed in PET-CT imaging to detect CRC *in vivo*. Combined with previous reports of elevated CDCP1 in CRC patient cohorts [3, 26], our results suggest that clinical implementation of a CDCP1-directed PET-CT imaging agent could have utility in CRC including for staging and assessment of treatment response as an aid to existing modalities of CT, MR, and FDG PET-CT imaging.

We have previously shown that antibody-based CDCP1 directed agents are effective for PET-CT imaging and treatment of preclinical models of ovarian [25] and pancreatic [24] cancer. The present study demonstrates the utility of a^89^Zr-labelled CDCP1-directed agent (10D7) for CRC and also extends the ovarian and pancreatic cancer studies by demonstrating the selectivity of ^89^Zr-10D7 for CDCP1 expressing tumors using unlabelled 10D7. The addition of high dose, unlabelled ligands is a recognized method to demonstrate *in vivo* target-specific blocking [41–43]. Our results indicate that unlabelled10D7 competes with ^89^Zr-10D7 for CDCP1 binding sites, thereby reducing PET signal by ∼60%, comparable to similar experimental design in existing publications [42, 44]. CRC13-shCDCP1 tumors had ∼40% reduced tumor avidity compared to CRC13-shSrc tumors. Comparable reductions are seen with other published silencing models [45]. Additionally, the peak avidity of CRC13-shCDCP1 tumors administered ^89^Zr-10D7 and nontransfected CRC13 administered ^89^Zr-IgG1*κ* was comparable. Residual radioligand uptake from silenced and nonspecific ligand experiments is attributed to nonspecific enhanced permeability (EPR) and retention effect [43], with exaggerated effects in most rapidly growing solid tumors [46] and *in vivo* small animal xenograft tumor models [47].

An underlying principle of radioligand molecular imaging is the provision of the lowest effective dose while maintaining diagnostically adequate spatial resolution [48]. The half-life of the chosen radionuclide must approximate that of the selected ligand [19]. ^89^Zr has a long half-life of 78.4 hours, making it suitable for full-length antibodies, such as 10D7 [19, 39]. However, the prolonged activity of such radionuclides results in increased off-target exposure [49]. As experienced in the *in vivo* models in this study, late imaging time points following the administration of a radiolabelled antibody are required to detect peak tumor-to-background avidity, at the expense of increased radiation exposure [19]. Peak *in vivo*^89^Zr-10D7 tumor-to-background signal was detected at 144 hours. Antibodies appeal as ligands because of high target specificity and affinity [50]. However, ligand serum half-life is dependent on size and structure [19, 39], with the relatively high molecular weight of ∼150 kDa of full-length antibodies such as 10D7 [24] requiring several days to reach peak tumor-to-background signal ratio due to initial blood pool and slower perfusion times compared to smaller vectors [40, 50–52]. The delay in achieving adequate tumor-to-background signal after agent administration may negatively impact clinical implementation of an antibody-based CDCP1-directed PET imaging agent for CRC and other cancers.

Another factor impacting implementation of a ^89^Zr-labelled antibody against CDCP1 is *in vivo* radionuclide metabolism and dissociation which results in 5–10% of conjugated ^89^Zr dissociating within 48 hours after administration. This is a clinical issue because unbound ^89^Zr accumulates in radiosensitive bone and skeletal growth plates, reducing its diagnostic utility and increasing bone marrow radiation dose [39, 40]. Another issue is the phenomena of *in vivo* transmetallation or transchelation of ^89^Zr with metal complexing proteins such as transferrin and ceruloplasmin in the liver and kidneys which also increases off-tumor irradiation [49].

These issues may be addressed by employing smaller CDCP1-targeted ligands including antibody fragments or peptides. Reducing the molecular size of full-length antibodies by altering the Fc receptor-binding domain can accelerate peak tumor-to-background ratio and improve blood clearance, tumor retention, and PET avidity [15]. Antibody fragments and peptides are smaller ligands with lower molecular weights and reduced serum half-lives which can more easily perfuse tissue [20, 39]. Antibody fragments typically share the same high-affinity binding and specificity properties as full-length counterparts [50] and some peptides also exhibit similar binding affinities as full-length antibodies with the added advantage of rapid tissue penetration [50, 52]. Peptides can also be resistant to protease hydrolysis, increasing *in vivo* stability [52]. In addition, low molecular weight ligands such as peptides are suited for radionuclides with short half-lives including ^18^F (half-life 109.8 min) and ^68^ Ga (half-life 67.6 min) [19, 39, 50]. Such radionuclides are favoured for radioligand molecular imaging as imaging can be performed soon after administration in a clinically manageable timeframe with reduced effective dose and minimal residual radioactivity on patient discharge [53, 54].

## 5. Conclusions

In summary, the preclinical murine models in this paper support CDCP1 as a radioligand molecular imaging target for CRC. Quantitative analysis by flow cytometry and semiquantitative analysis by immunohistochemistry confirm CDCP1 expression in a range of CRC cell lines and a PDX. Statistically significant high tumor uptake of the radio-labelled antibody ^89^Zr-10D7 was found in two CDCP1-expressing CRC mouse models at late imaging time points with specificity indicated on protein silencing and epitope blocking models. The findings support further work to examine CDCP1 as a radioligand molecular imaging target for CRC.

## Data Availability

The data used to support the findings of this study are available from the corresponding author upon request.

## Supplementary Materials

Supplementary Materials Table S1: two-way ANOVA of statistically significant *in vivo* PET avidity (%ID/cc) of HCT 116 tumors for imaging time points. Table S2: *ex vivo* radiometric gamma analysis activity (%ID/g mean ± SD) of HCT116 tumors at 144 hours. Table S3: two-way ANOVA of statistically significant *ex vivo* radiometric gamma analysis activity (%ID/g) of HCT116 tumors at 144 hours. Table S4: *ex vivo* radiometric gamma analysis activity (%ID/g mean ± SD) of CRC13 at 144 hours.
