# Supplementary material for: Preclinical Molecular PET-CT Imaging Targeting CDCP1 in Colorectal Cancer
Source: Contrast Media Mol Imaging. 2021 Sep 13;2021:3153278. doi: 10.1155/2021/3153278 (PMC8455202; doi:10.1155/2021/3153278)
Supplement: Supplementary Materials — Table S1: two-way ANOVA of statistically significant in vivo PET avidity (%ID/cc) of HCT 116 tumors for imaging time points. Table S2: ex vivo radiometric gamma analysis activity (%ID/g mean ± SD) of HCT116 tumors at 144 hours. Table S3: two-way ANOVA of statistically significant ex vivo radiometric gamma analysis activity (%ID/g) of HCT116 tumors at 144 hours. Table S4: ex vivo radiometric gamma analysis activity (%ID/g mean ± SD) of CRC13 at 144 hours. [file 3153278.f1.docx]

## SUPPLEMENTARY TABLES

**Table S1: Two-way ANOVA of statistically significant *in vivo* PET avidity (%ID/cc) of HCT 116 tumors for imaging time points**

| **24 hours** | **p-value** |
| --- | --- |
| ^89^Zr-10D7 vs. ^89^Zr-IgG1κ | <0.001 |
| ^89^Zr-IgG1κ vs. Unlabelled 10D7 & ^89^Zr-10D7 | <0.05 |

| **48 hours** | **p-value** |
| --- | --- |
| ^89^Zr-10D7 vs. ^89^Zr-IgG1κ | <0.001 |
| ^89^Zr-10D7 vs. Unlabelled 10D7 & ^89^Zr-10D7 | <0.001 |
| ^89^Zr-IgG1κ vs. Unlabelled 10D7 & ^89^Zr-10D7 | <0.001 |

| **72 hours** | **p-value** |
| --- | --- |
| ^89^Zr-10D7 vs. ^89^Zr-IgG1κ | <0.001 |
| ^89^Zr-10D7 vs. Unlabelled 10D7 & ^89^Zr-10D7 | <0.001 |
| ^89^Zr-IgG1κ vs. Unlabelled 10D7 & ^89^Zr-10D7 | <0.001 |

| **144 hours** | **p-value** |
| --- | --- |
| ^89^Zr-10D7 vs. ^89^Zr-IgG1κ | <0.001 |
| ^89^Zr-10D7 vs. Unlabelled 10D7 & ^89^Zr-10D7 | <0.001 |
| ^89^Zr-IgG1κ vs. Unlabelled 10D7 & ^89^Zr-10D7 | <0.001 |

**Table S2: *Ex vivo* radiometric gamma analysis activity (%ID/g mean ± SD) of HCT116 tumors at 144 hours**

| **Tissue** | **^89^Zr-10D7** | **Unlabelled 10D7 & ^89^Zr-10D7** | **^89^Zr-IgG1κ** |
| --- | --- | --- | --- |
| Tumour (HCT116) | 13.8 ± 2.6 | 3.1 ± 0.8 | 1.2 ± 0.3 |
| Heart | 1.8 ± 0.1 | 3.2 ± 0.6 | 1.1 ± 0.1 |
| Lungs | 4.3 ± 0.8 | 6.2 ± 1.2 | 1.8 ± 0.3 |
| Kidneys | 2.1 ± 0.1 | 2.8 ± 0.2 | 1.7 ± 0.2 |
| Femur | 3.4 ± 0.3 | 3.4 ± 0.7 | 8.5 ± 3.1 |
| Muscle | 0.6 ± 0.1 | 1.0 ± 0.1 | 0.3 ± 0.1 |
| Liver | 13.9 ± 0.8 | 17.5 ± 0.9 | 24.7 ± 2.7 |
| Tail | 1.3 ± 0.2 | 1.5 ± 0.2 | 1.2 ± 0.1 |
| Blood | 5.0 ± 0.6 | 7.6 ± 0.8 | 0.5 ± 0.0 |
| Testes | 1.3 ± 0.2 | 1.9 ± 0.4 | 1.6 ± 0.2 |

**Table S3: Two-way ANOVA of statistically significant *ex vivo* radiometric gamma analysis activity (%ID/g) of HCT116 tumors at 144 hours**

| **Tumour** | **p-value** |
| --- | --- |
| ^89^Zr-10D7 vs. ^89^Zr-IgG1κ | <0.05 |
| ^89^Zr-10D7 vs. Unlabelled 10D7 & ^89^Zr-10D7 | <0.0001 |
| ^89^Zr-IgG1κ vs. Unlabelled 10D7 & ^89^Zr-10D7 | <0.0001 |

| **Heart** | **p-value** |
| --- | --- |
| ^89^Zr-10D7 vs. ^89^Zr-IgG1κ | ns |
| ^89^Zr-10D7 vs. Unlabelled 10D7 & ^89^Zr-10D7 | ns |
| ^89^Zr-IgG1κ vs. Unlabelled 10D7 & ^89^Zr-10D7 | <0.05 |

| **Lungs** | **p-value** |
| --- | --- |
| ^89^Zr-10D7 vs. ^89^Zr-IgG1κ | <0.01 |
| ^89^Zr-10D7 vs. Unlabelled 10D7 & ^89^Zr-10D7 | <0.05 |
| ^89^Zr-IgG1κ vs. Unlabelled 10D7 & ^89^Zr-10D7 | <0.0001 |

| **Kidneys** | **p-value** |
| --- | --- |
| ^89^Zr-10D7 vs. ^89^Zr-IgG1κ | ns |
| ^89^Zr-10D7 vs. Unlabelled 10D7 & ^89^Zr-10D7 | ns |
| ^89^Zr-IgG1κ vs. Unlabelled 10D7 & ^89^Zr-10D7 | ns |

| **Femur** | **p-value** |
| --- | --- |
| ^89^Zr-10D7 vs. ^89^Zr-IgG1κ | <0.0001 |
| ^89^Zr-10D7 vs. Unlabelled 10D7 & ^89^Zr-10D7 | ns |
| ^89^Zr-IgG1κ vs. Unlabelled 10D7 & ^89^Zr-10D7 | <0.0001 |

| **Muscle** | **p-value** |
| --- | --- |
| ^89^Zr-10D7 vs. ^89^Zr-IgG1κ | ns |
| ^89^Zr-10D7 vs. Unlabelled 10D7 & ^89^Zr-10D7 | ns |
| ^89^Zr-IgG1κ vs. Unlabelled 10D7 & ^89^Zr-10D7 | ns |

| **Liver** | **p-value** |
| --- | --- |
| ^89^Zr-10D7 vs. ^89^Zr-IgG1κ | <0.0001 |
| ^89^Zr-10D7 vs. Unlabelled 10D7 & ^89^Zr-10D7 | <0.0001 |
| ^89^Zr-IgG1κ vs. Unlabelled 10D7 & ^89^Zr-10D7 | <0.0001 |

| **Tail** | **p-value** |
| --- | --- |
| ^89^Zr-10D7 vs. ^89^Zr-IgG1κ | ns |
| ^89^Zr-10D7 vs. Unlabelled 10D7 & ^89^Zr-10D7 | ns |
| ^89^Zr-IgG1κ vs. Unlabelled 10D7 & ^89^Zr-10D7 | ns |

| **Blood** | **p-value** |
| --- | --- |
| ^89^Zr-10D7 vs. ^89^Zr-IgG1κ | <0.01 |
| ^89^Zr-10D7 vs. Unlabelled 10D7 & ^89^Zr-10D7 | <0.0001 |
| ^89^Zr-IgG1κ vs. Unlabelled 10D7 & ^89^Zr-10D7 | <0.0001 |

| **Testes** | **p-value** |
| --- | --- |
| ^89^Zr-10D7 vs. ^89^Zr-IgG1κ | ns |
| ^89^Zr-10D7 vs. Unlabelled 10D7 & ^89^Zr-10D7 | ns |
| ^89^Zr-IgG1κ vs. Unlabelled 10D7 & ^89^Zr-10D7 | ns |

ns, not significant

**Table S4: *Ex vivo* radiometric gamma analysis activity (%ID/g mean ± SD) of CRC13 at 144 hours**

| **Tissue** | **^89^Zr-10D7** | **^89^Zr-IgG1κ** |
| --- | --- | --- |
| Tumour (CRC13) | 13.1 ± 1.7 | 4.9 ± 0.8 |
| Heart | 3.5 ± 0.3 | 1.6 ± 0.2 |
| Lungs | 5.7 ± 0.5 | 5.5 ± 8.2 |
| Kidneys | 2.3 ± 0.4 | 1.5 ± 0.7 |
| Femur | 3.5 ± 0.9 | 6.5 ± 1.1 |
| Muscle | 1.1 ± 0.2 | 0.7 ± 0.2 |
| Liver | 5.1 ± 0.7 | 13.4 ± 1.9 |
| Tail | 1.7 ± 0.4 | 2.2 ± 0.5 |
| Blood | 9.8 ± 2.0 | 2.0 ± 0.3 |
| Testes | 1.8 ± 0.3 | 1.3 ± 0.8 |
